# Laparoscopic Removal of Angelchik Prosthesis Followed by Interval Sleeve Gastrectomy

**DOI:** 10.1155/2019/2479267

**Published:** 2019-05-21

**Authors:** Faryal G. Afridi, Morgan Johnson, Kelsey A. Musgrove, Salim Abunnaja, Lawrence E. Tabone, David C. Borgstrom, Nova Szoka

**Affiliations:** Department of Surgery, West Virginia University, Morgantown, USA

## Abstract

**Introduction:**

The Angelchik prosthesis (AP) is a historic antireflux device which consists of a C-shaped silicone ring placed around the gastroesophageal junction (GEJ) and secured by Dacron tape. We present a rare experience with an AP and its impact on bariatric surgical outcomes.

**Case:**

Our patient is a 66-year-old woman who had an open antireflux procedure with an AP in 1987. She presented to a bariatric clinic for consideration of bariatric surgery for the treatment of morbid obesity and associated comorbidities. She also reported significant problems with reflux and dysphagia. After an appropriate work-up, an AP was identified at her GEJ. She was taken to the operating room for laparoscopic removal with planned interval laparoscopic sleeve gastrectomy. Intraoperatively, the AP was identified around the GEJ; after extensive adhesiolysis, the prosthesis was removed. Postoperatively, in order to determine if the AP had caused any lasting esophageal motility problems, the patient underwent a high-resolution esophageal manometry which demonstrated normal esophageal motility. Interval laparoscopic sleeve gastrectomy was performed safely 9 weeks later.

**Conclusion:**

Although rarely used, it is still possible to encounter an Angelchik prosthesis in practice. General and bariatric surgeons need to be aware of this rare device and understand how to manage its related complications.

## 1. Introduction

The Angelchik prosthesis (AP) was first introduced in 1979 as an antireflux device and initially became popular due to its simple design and ease of insertion [1–3]. It is a C-shaped silicon ring that is fitted around the gastroesophageal junction (GEJ) and secured in place by a Dacron ribbon [2]. However, it is a device rarely seen today due to widespread reports of complications such as migration, displacement, erosion, and dysphasia [1, 2]. Displacement may be directed upward into the mediastinum through a wide hiatus, downward onto the body of the stomach, or by a 180-degree rotation pinching the esophagus. The most common of these complications was dysphagia (reported rates of 70% up to 96%), followed by device migration and device erosion. [1, 2] Incidence of postoperative dysphagia increased over time, with initial results showing significant rates of dysphagia (37% to 43% at 1 year), with rates only increasing with longer follow up [4–6]. Dysphagia was noted to be directly caused by the prosthesis, demonstrated by greatly delayed esophageal emptying times [7]. Removal was required most frequently due to dysphagia or reflux, and the rate of a second operation was 10% or greater, a rate much higher than other antireflux operations [7–9]. Due to these results, the device became obsolete as a routine option for the treatment of gastroesophageal reflux disease. However, it is estimated that more than 30,000 Angelchik devices were implanted worldwide prior to this, and thus it is still possible to encounter these devices in current surgical practice [10].

## 2. Case Summary

Our patient is a 66-year-old woman referred to a bariatric clinic for consideration of bariatric surgery for long-term morbid obesity with BMI of 44 and associated comorbidities including diabetes, sleep apnea, and GERD. She had a surgical history of an open antireflux procedure with an Angelchik prosthesis in 1987. She reported significant problems with daily reflux and progressive dysphagia to solids and some liquids. Her initial work-up included an upper GI study, an esophagogastroduodenoscopy (EGD), and a CT scan. The upper GI study and CT scan showed the AP in place at the GEJ. On retroflexion during the EGD, the indentation of a band of foreign body was present at the GEJ without evidence of erosion.

She underwent elective laparoscopic removal of the AP, with plan for interval laparoscopic sleeve gastrectomy. During the operation to remove the AP, dense adhesions were encountered between the site of the device, the fundus of the stomach, diaphragm, and the left lobe of the liver.

**Figure 1:**
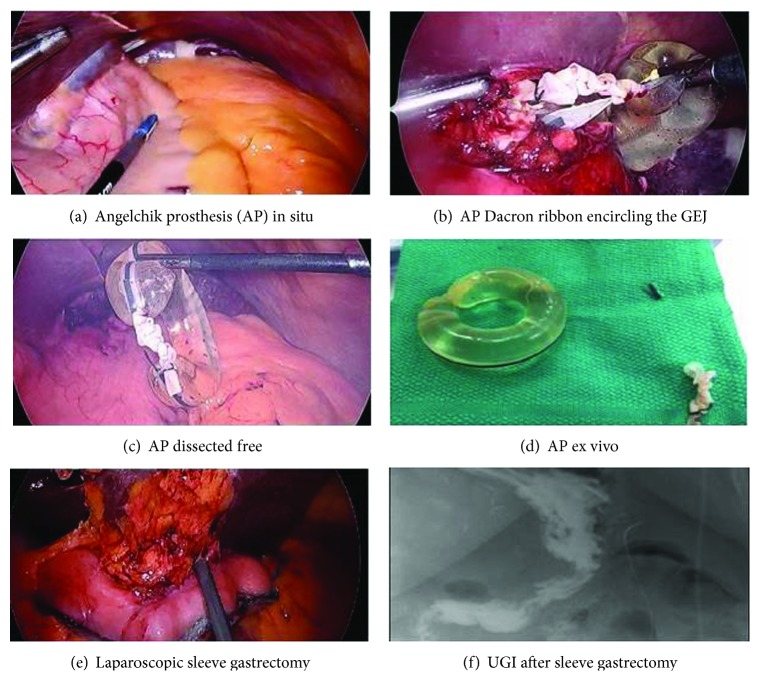
(a–f) Laparoscopic removal of Angelchik prosthesis and interval sleeve gastrectomy. (a) Angelchik prosthesis (AP) in situ. (b) AP Dacron ribbon seen encircling the gastroesophageal junction (GEJ). (c) AP dissected free. (d) Angelchik prosthesis ex vivo. (e) Laparoscopic sleeve gastrectomy. (f) UGI after laparoscopic sleeve gastrectomy.

After performing extensive adhesiolysis, the AP was visualized and removed (Figures 1(a)–1(d)). Postoperatively, her dysphagia and reflux was resolved. In order to determine if the AP had caused any lasting esophageal motility problems, she underwent a high-resolution esophageal manometry which demonstrated normal motility.

After this work-up and recovery, laparoscopic sleeve gastrectomy was safely performed nine weeks later (Figure 1(e)). Due to significant adhesive disease, the left crus was not well visualized. At her 3-month follow-up visit, the patient had lost 9 lbs since surgery (10% EWL) and she continued to remain free of dysphagia or reflux symptoms. An upper GI series demonstrated retained fundus, which may explain the inadequate weight loss (Figure 1(f)).

## 3. Discussion

Although the Angelchik prostheses are no longer being used, it is still possible to encounter one in practice secondary to the myriad of complications associated with them. General and bariatric surgeons should be aware of this rare device and understand how to manage its related complications. Stewart et al. in 1994 described their outcomes after removal of the device secondary to dysphagia, recurrent reflux, and migration [11]. They noted frequent intraoperative difficulty with removal due to a dense fibrous capsule [11]. Another case report of laparoscopic removal of the Angelchik prosthesis due to complaints of dysphagia also noted dense adhesions to the proximal stomach, diaphragm, and liver with an extensive fibrous pseudocapsule enclosing the ring. An UGI postoperatively showed no evidence of contrast extravasation and the patient was discharged in 2 days with resolution of dysphagia [10].

It has also been observed that if reflux was a dominant complaint prior to Angelchik prosthesis placement, reflux was commonly noted to recur after the removal of the device [11]. It was suggested by Stewart et al. that an alternative antireflux procedure be performed in these cases [11]. Our patient's dysphagia and reflux was resolved after removal, and interval high-resolution manometry revealed normal esophageal motility. Thus, laparoscopic sleeve gastrectomy was performed in order to address the patient's remaining issue of severe morbid obesity and associated comorbidities. Laparoscopic sleeve gastrectomy is effective in producing weight loss and improving diabetes; however, evidence of the effect on GERD postoperatively has been inconsistent [12, 13]. Therefore for patients with persistent reflux preoperatively after removal of an Angelchik device, it may be prudent to discuss alternative antireflux options at the time of surgery. In addition, in patients similar to ours with concomitant morbid obesity, a Roux-en-Y gastric bypass as an alternative to sleeve gastrectomy can more effectively address the reflux symptoms. Although there is no literature available to support whether to perform laparoscopic sleeve gastrectomy or laparoscopic Roux-en-Y gastric bypass in patients who undergo removal of an Angelchik prosthesis, an AP is a foreign body around the GEJ with similar characteristics to a laparoscopic gastric band. Data suggest that conversion from a gastric band to either laparoscopic sleeve gastrectomy or laparoscopic Roux-en-Y gastric bypass is safe and efficacious when performed in a single-stage or a delayed fashion. The decision to convert this patient to a sleeve gastrectomy instead of a gastric bypass was made for several reasons: first, the patient did not have ongoing dysphagia or reflux symptoms and had normal esophageal motility following AP removal; second, she had significant intraabdominal adhesive disease; and third, she had chronic lower extremity pain with possible ongoing need for nonsteroidal anti-inflammatory medications (NSAIDs). In order to be a safe candidate for gastric bypass, patients must commit to being NSAID-free lifelong, as the use of NSAIDs following gastric bypass can lead to marginal ulcer formation. For patients who have an AP removed but continue to have significant reflux or dysphagia symptoms and morbid obesity, conversion to a gastric bypass is the optimal approach to address both conditions.

Our case, as well as the previous literature, suggests that it is important to be aware of the pathophysiology of the Angelchik prosthesis, as patients may present with associated complications. There is relative difficulty with removal described in various case reports. Laparoscopic removal should therefore be performed by surgeons familiar with the esophageal hiatus. Intervention should be considered highly in patients presenting with any of the common symptoms, particularly dysphagia and reflux, as resolution of these is noted in the majority of case reports describing removal.

## Supplementary Materials

Supplementary Materials The attached case video demonstrates the case of a 66-year-old woman with a history of morbid obesity who presented to a bariatric surgery clinic with the complaint of dysphagia. Further work-up noted an Angelchik prosthesis at her gastroesophageal junction. She underwent staged removal of the prosthesis with resolution of her dysphagia and subsequent laparoscopic sleeve gastrectomy.
